# Perceptions of discharge readiness among primiparas based on the King’s theory of goal attainment: a qualitative study

**DOI:** 10.1590/1980-220X-REEUSP-2025-0187en

**Published:** 2025-12-15

**Authors:** Hangcheng Liu, Hong Jiang, Miao Li, Xixi Li

**Affiliations:** 1 University of Electronic Science and Technology of China, School of Medicine, Mianyang Central Hospital, Department of Obstetrics and Gynecology, Mianyang, Sichuan Province, China.; 2 University of Electronic Science and Technology of China, School of Medicine, Mianyang Central Hospital, Department of Nursing, Mianyang, Sichuan Province, China.; 3 North Sichuan Medical College, College of Nursing, Nanchong, Sichuan Province, China.

**Keywords:** Parity, Patient Discharge, Nurse-Patient Relations, Paridade, Alta do Paciente, Relações Enfermeiro-Paciente

## Abstract

**Objective:**

To analyze primiparas’ perceived experiences and unmet nursing needs regarding discharge readiness, using the King’s Theory of Goal Attainment, to optimize clinical discharge management.

**Methods:**

Using the phenomenological method in qualitative research, through purposive sampling, 18 primiparas from a tertiary hospital in Sichuan Province were subjected to face-to-face semi-structured in-depth interviews. NVivo 15.0 software was used to manage the interview data, and the Colaizzi phenomenological 7-step analysis method was used to analyze the data.

**Results:**

A total of 5 main themes and 12 sub-themes have been identified, including complex psychology at discharge (positive expectations, full of worries), promoting factors of discharge preparation (personal proactive coping, the degree of family support was high), hindering factors of discharge preparation (the self-care knowledge was weak, insufficient self-care skills, difficulties in parenting), nursing service needs (maternal and infant knowledge and nursing needs, continuing nursing knowledge needs), and interactive participation (the implementation of interactive assessments is inadequate, insufficient participation, expect to participate in decision-making).

**Conclusion:**

Primiparas’ discharge preparation needs remain unmet. Medical staff should address various factors affecting discharge readiness, enhance the interaction between nurses and patients, and increase the initiative of primiparas in self-management, to help them adapt to life after discharge and achieve maternal and infant health goals.

## RESUMO

**Objetivo:**

Analisar as experiências percebidas e as necessidades de enfermagem não atendidas de primíparas em relação à preparação para alta hospitalar, utilizando a Teoria do Alcance de Metas de King, para otimizar o gerenciamento clínico da alta.

**Métodos:**

Utilizando o método fenomenológico na pesquisa qualitativa, por meio de amostragem intencional, 18 primíparas de um hospital terciário na Província de Sichuan foram submetidas a entrevistas em profundidade semiestruturadas e face a face. O software NVivo 15.0 foi utilizado para gerenciar os dados das entrevistas, e o método de análise fenomenológica de 7 etapas de Colaizzi foi utilizado para analisar os dados.

**Resultados:**

Foram identificados um total de 5 temas principais e 12 subtemas, incluindo: psicologia complexa na alta (expectativas positivas, cheias de preocupações), fatores promotores da preparação para alta (enfrentamento proativo pessoal, alto grau de suporte familiar), fatores impeditivos da preparação para alta (conhecimento sobre autocuidado fraco, habilidades de autocuidado insuficientes, dificuldades na parentalidade), necessidades de serviço de enfermagem (necessidades de conhecimento e cuidados materno-infantis, necessidades de conhecimento sobre continuidade dos cuidados) e participação interativa (a implementação de avaliações interativas é inadequada, participação insuficiente, expectativa de participar na tomada de decisões).

**Conclusão:**

As necessidades de preparação para alta das primíparas permanecem não atendidas. A equipe médica deve abordar os diversos fatores que afetam a prontidão para alta, melhorar a interação entre enfermeiros e pacientes e aumentar a iniciativa das primíparas no autocuidado, para ajudá-las a adaptar-se à vida após a alta e alcançar os objetivos de saúde materno-infantil.

## INTRODUCTION

The World Health Organization (WHO) indicates that approximately 260,000 maternal deaths occurred globally in 2023 during pregnancy, childbirth, or the postpartum period, with the majority of these deaths being preventable^(1)^, highlighting the crucial need for maternal safety. In 2021, the Chinese Optimizing Fertility Policies to Promote Long-Term Balanced Population Development emphasized the full implementation of the advancement of integrated postpartum care services for mothers^(2)^. Concurrently, China’s healthcare reforms and widespread adoption of enhanced recovery protocols have transformed perinatal care models^(3,4)^. While benefiting mothers through faster functional recovery, pain relief, shorter hospital stays, and lower costs, these changes risk premature discharge before full physiological recovery, potentially increasing postpartum physical and psychological adaptation challenges^(5,6)^.

Readiness for Hospital Discharge (RHD), introduced by British scholar Fenwick in 1979, involves a multidimensional assessment (physiological, psychological, and social functional) by healthcare providers to determine a patient’s ability to transition from hospital to home^(7)^. As a key element of discharge planning, RHD has been widely implemented in clinics^(8)^. Research confirms that optimal discharge readiness reduces readmission risks, prevents post-discharge complications, and enhances physiological stability^(9)^. Insufficient discharge readiness may lead to a lack of relevant knowledge and skills of primiparas, temporary lack of self-care ability and infant care ability, which can easily produce postpartum anxiety, depression, and other psychological problems, which will affect the maternal rehabilitation, and even seriously affect the growth and development of infants^(6)^. Primiparous women are more vulnerable to pregnancy complications due to their first experience with gestation and delivery. Factors like limited health knowledge, lack of nursing skills, poor psychological adaptability, and shorter hospital stays contribute to their higher risk^(10)^.

**Figure 1:**
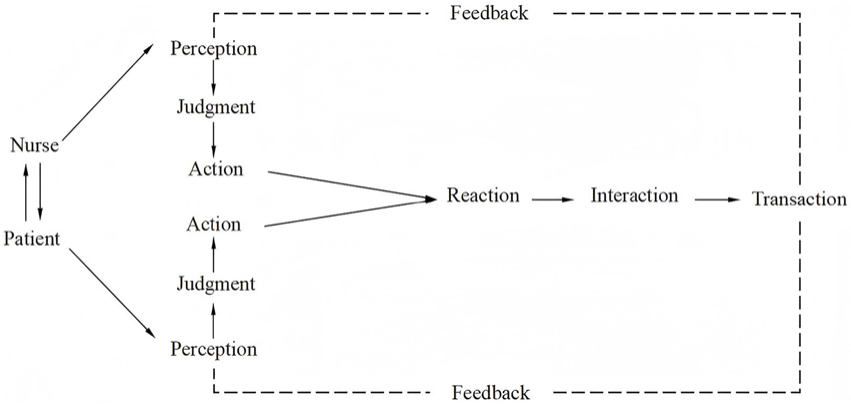
King’s theory of goal attainment.

The King’s Theory of Goal Attainment (TGA) was proposed by King in 1981, which emphasizes “patient-centered” care in the nursing process and encourages patients to actively participate in health care. Through the four-stage cycle model of “nurse-patient interaction, goal synergy, measure, and dynamic evaluation”, patients can be promoted to reach the best state in a short period^(11)^. This process demonstrates the systematic and dynamic characteristics of nursing activities, as shown in Figure 1. This theory has been widely used in the fields of chronic disease management, health education, psychological nursing, and other fields and has been proven to improve the health outcomes of patients^(12)^.

Therefore, based on the King’s TGA and the phenomenological research method in qualitative research, this study conducted in-depth interviews with primiparas to clarify their multi-dimensional perceived experience of discharge readiness, identify their unmet nursing needs, and provide a basis for clinical medical and nursing staff to optimize the management of discharge readiness of primiparas.

## METHOD

### Design and Setting

This study adopted a qualitative design. The phenomenological research method^(13)^ was used to analyze primiparas’ perception of discharge preparation and nursing needs. The results of this study were reported using the Unified Standards for Qualitative Research Reporting (COREQ)^(14)^.

### Population, Inclusion, and Exclusion Criteria

Using purposive sampling, we selected postpartum women who visited the obstetrics ward of a tertiary Grade A general hospital in Sichuan Province between April and May 2024 as study participants. The sampling strategy followed maximum variation sampling to ensure diversity across multiple dimensions, including maternal age, educational level, occupation, and place of residence. Maternal inclusion criteria were: 1) age ≥ 20 years old^(15)^; 2) primipara; 3) the postpartum condition was stable and discharged smoothly; 4) normal mental consciousness and good communication ability; 5) those who voluntarily participate and sign informed consent. The exclusion criteria were as follows: 1) severe comorbidities/complications before or after delivery; 2) abnormality of newborns; 3) those who withdrew voluntarily during the interview.

### Interview and Procedure

The researchers reviewed the relevant literature at home and abroad, guided by the King’s TGA and combined with the suggestions of relevant clinical experts, the research team (including 1 master’s supervisor, 6 graduate students, 1 deputy chief physician in obstetrics, and 1 deputy chief nurse in obstetrics) initially drew up an interview outline. Three primiparas who met the inclusion and exclusion criteria were pre-interviewed, and the interview outline was revised according to the interview results. The formal interview outline is shown in Chart 1.

**Chart 1:** Interview guide – Mianyang, Sichuan, China, 2024.

Number	Questions
1	As you prepare to go home from the hospital, can you describe how you feel right now? (including physical and mental state)
2	What have you learned about your own rehabilitation and infant care in order to successfully move from hospital to home care?
3	Did you have any problems during your stay? If so, how was your problem resolved?
4	What kind of guidance did the nurse give you during your hospitalization?
5	During discharge preparation, were you involved in the decision-making process? (e.g., discharge time, care planning, etc.) What do you think of this level of involvement?
6	Do you have any suggestions or comments to help you discharge from the hospital to your home?

This study used a phenomenological approach to analyze primiparas’ perceptions of discharge readiness and care needs. Before data collection, all researchers received systematic training in qualitative methodologies. With introductions from obstetricians or charge nurses, researchers established initial rapport with potential participants, followed by detailed explanations of study objectives, procedures, confidentiality measures, and voluntary participation principles, culminating in written informed consent. Interviews were scheduled within 24 hours before discharge. A private space (single-bed room or family-centered care unit) was selected through mutual agreement to ensure confidentiality. Semi-structured interviews, averaging 30–40 minutes in length, were audio-recorded. Researchers adhered to the interview guide while maintaining flexibility, practicing active listening, documenting non-verbal cues (e.g, facial expressions, tone shifts), and avoiding leading questions or interruptions. For data management, all materials were anonymized (P1–P20) and stored encrypted by the principal investigator. Verbatim transcription and reflexive journaling were completed within 24 hours post-interview. The final sample included 18 Primiparas (initial recruitment: 20; 2 withdrew due to infant care needs), with sampling continuing until data saturation.

### Data Analysis

Within 24 hours after the interview, the researcher promptly transcribed the recording into text data, supplemented the non-verbal information, such as movements and expressions recorded during the interview, and then handed the recording data and text data to another trained research team member for verification to ensure the completeness and accuracy of the transcription. In this study, NVivo 15.0 software was used to manage the text data, and the Colaizzi phenomenological 7-step analysis method^(16)^ was used to analyze the data. The specific steps are as follows: (1) Repeatedly listen to and read the collected materials, familiarize yourself with all the content, and form an overall understanding; (2) Import the Word text materials into the NVivo 15.0 software, read them word by word, identify and extract the relevant and meaningful statements that are related to the research question; (3) Code the repetitive and meaningful statements; (4) Summarize and categorize the coded viewpoints, and construct a thematic framework; (5) Describe each theme in detail and insert typical original statements; (6) Identify similar viewpoints and determine the themes; (7) Feedback the results to the respondents to verify the authenticity and accuracy of the content.

### Ethical Approval

The study was conducted in strict accordance with relevant guidelines and regulations and the principles of the Declaration of Helsinki. The study was approved by the Biomedical Ethics Committee of Mianyang Central Hospital, University of Electronic Science and Technology of China, before the study was conducted (ID: S20240310-02). Before the interview, the researcher fully informed the primipara about the content of the study and signed an informed consent. Transcripts were anonymized by replacing names with codes (P1–P18). Participants could withdraw from the study at any time.

## RESULTS

### Demographic Characteristics of Participants

According to the principle of data saturation, a total of 18 married primiparas who met the inclusion and exclusion criteria were included as interviewees. According to the principle of confidentiality, the name of the interviewee object was replaced by the number, namely, P1–P18. The general information of the interviewees is shown in Table 1.

**Table 1:** General information about the interviewees (n = 18) – Mianyang, Sichuan, China, 2024.

Number	Age (year)	Ethnic groups	Education background	Occupations	Place of residence	Per capita income (yuan/month)	Medical insurance	Comorbidity	Mode of delivery	Caregiver(s)
P1	24	Han	Junior college	–	Town of the county	3000-5000	New rural cooperative medical system	–	Natural birth	1
P2	28	Han	Bachelor's degree	Staff member	Urban area	>8000	Medical treatment for workers	–	Cesarean section	2
P3	28	Han	Junior college	Staff member	Urban area	5000-8000	Medical treatment for workers	–	Natural birth	1
P4	31	Han	Technical secondary school	–	Urban area	5000-8000	New rural cooperative medical system	GDM, ICP	Cesarean section	2
P5	36	Qiang	Bachelor's degree	Civil service	Urban area	>8000	Medical treatment for workers	GDM, Hyperthyroidism	Cesarean section	2
P6	28	Han	Junior college	Teacher	Urban area	3000-5000	Medical treatment for workers	–	Cesarean section	1
P7	27	Han	Technical secondary school	–	Urban area	5000-8000	New rural cooperative medical system	–	Cesarean section	2
P8	34	Han	Bachelor's degree	Teacher	Urban area	>8000	Medical treatment for workers	–	Cesarean section	2
P9	39	Han	Junior college	Teacher	Urban area	5000-8000	Medical treatment for workers	–	Cesarean section	1
P10	29	Han	Junior college	Staff member	Urban area	5000-8000	Medical treatment for workers	–	Cesarean section	2
P11	33	Han	Master's degree	Nurse	Urban area	>8000	Medical treatment for workers	Thrombophilia	Cesarean section	2
P12	27	Han	Bachelor's degree	Nurse	Urban area	>8000	Medical treatment for workers	–	Natural birth	1
P13	36	Han	Junior Middle School	–	Township	3000-5000	New rural cooperative medical system	–	Cesarean section	1
P14	22	Han	High school	Staff member	Township	3000-5000	New rural cooperative medical system	–	Natural birth	1
P15	29	Han	Master's degree	Staff member	Urban area	>8000	Medical treatment for workers	–	Cesarean section	2
P16	27	Tujia	Junior college	–	Town of the county	5000-8000	Out of pocket	–	Natural birth	2
P17	29	Han	Bachelor's degree	Civil service	Urban area	5000-8000	Medical treatment for workers	Hypothyroidism	Cesarean section	1
P18	26	Han	High school	–	Urban area	5000-8000	New rural cooperative medical system	GDM	Cesarean section	2

Note. GDM, gestational diabetes mel I itus; ICP, intrahepatic cholestasis of pregnancy.

Eighteen recordings were transcribed verbatim, and 12 sub-themes with similar concepts were finally formed through manual coding, clustering themes, and repeated deliberation. 12 sub-themes were analyzed and summarized into five themes: complex psychology at discharge, promoting factors of discharge preparation, hindering factors of discharge preparation, nursing service needs, and interactive participation, as shown in Chart 2.

**Chart 2:** Key themes and sub-themes – Mianyang, Sichuan, China, 2024.

Themes	Sub-Themes
Complex psychology at discharge	1. Positive expectations
	2. Full of worries
Promoting factors of discharge preparation	1. Personal proactive coping
	2. The degree of family support was high
Impediments to discharge preparation	1. The self-care knowledge was weak
	2. Insufficient self-care skills
	3. Difficulties in parenting
Nursing service needs	1. Maternal and infant knowledge and nursing needs
	2. Continuing nursing knowledge needs
Interactive participation	1. The implementation of interactive assessments is inadequate
	2. Insufficient participation
	3. Expect to participate in decision-making

### Theme 1: Complex Psychology at Discharge

#### Sub-Theme 1: Positive Expectations

Some primiparas believe that they have recovered well and have no postpartum complications, and with their families providing sufficient care and support, they are eager to be discharged from the hospital. Others, however, due to the relatively unfamiliar, noisy hospital environment, lack of privacy protection, and irregular schedules, are more eager to return to the familiar and comfortable home environment.

*The whole process of childbirth went well (laughs). I’m happy to go home. (P1)*

*The ward was so narrow that it was easy for the babies to affect each other, one crying, the other crying, and worrying about disturbing others. The home is more spacious, so the two people who accompany the bed can take turns to rest, which is more convenient. (P5)*

*The moment I was discharged from the hospital, I was very happy, because I had been staying up all night and felt so tired. (P12)*

*The mood is very happy, the bed in the ward is too hard, and the environment is better after going home. (P17)*

#### Sub-Theme 2: Full of Worries

Due to the shortened hospital stay and being a first-time mother, some primiparas mainly worry about poor recovery after discharge, insufficient ability to care for the newborn, lack of professional medical support, and concerns about potential risks.

*Worried about how to get home, afraid to walk because the wound is still very painful (frown). The baby was in the care unit, and I was worried about breastfeeding because my breasts swelled easily, and I didn’t know what to do. (P2)*

*I worry that I can’t take good care of the baby when I get home. (P6)*

*I worry about whether I’ll have trouble defecating when I get home. Also worried that the baby’s jaundice is unusual, and feeding is difficult. (P12)*

*I feel the wound is still relatively painful, afraid the wound recovery is not good. (P18)*

### Theme 2: Promoting Factors of Discharge Preparation

#### Sub-Theme 1: Personal Proactive Coping

In the information age, with the help of network intelligence, puerpera take the initiative to obtain maternal and child care support by consulting medical staff, joining mother groups, searching parenting knowledge, and reading professional books.

*With the guidance of the midwife, I learned how to breastfeed. (P3)*

*I learned rehabilitation videos, such as the pelvic floor muscle and the rectus abdominis muscle, on TikTok. I also attended a maternity communication group at the hospital to learn about diaper choices and how to manage your baby’s eczema and vomiting. (P4)*

*I bought a book about the postpartum diet for study. (P10)*

*If I encounter problems, I can ask my friends who have gone through similar situations and learn from their experiences. (P15)*

#### Sub-Theme 2: The Degree of Family Support was High

In the special stage of new motherhood, primiparas received comprehensive support and care from partners, friends, and family, which not only reduced their parenting burden but also allowed them more time to rest and recover, thus enhancing their parenting sense of competence and preparing them for various challenges after discharge.

*My mood is good, they (husband and mother) take good care of him (baby), they have been studying these days, and I believe they will do very well. (P1)*

*In terms of babies, my sister has two children. Both my sister and my mother are experienced enough to know about the care and feeding of babies, and my recovery and precautions. (P2)*

*The observation, bathing, and touching of the baby’s jaundice are all being learned by the father. (P6)*

### Theme 3: Impediments to Discharge Preparation

#### Sub-Theme 1: The Self-Care Knowledge was Weak

Due to the influence of modern parenting concepts, convenient service, and family support, some primiparas have insufficient cognition of the complexity of postpartum recovery and child care, which can easily produce dependence, low willingness to self-learning, and a general lack of postpartum rehabilitation and child care knowledge.

*Once I’m back home, my family handles everything, so I can just focus on resting. (P1)*

*I don’t have any experience. I go to the postnatal care center to learn. (P9)*

*Because I hired my postpartum doula, I now have a lot of willpower, after going home to study slowly. (P12)*

#### Sub-Theme 2: Insufficient Self-Care Skills

Postpartum women are in a recovery period, and fatigue and discomfort will limit their time and energy for learning self-care skills. Moreover, due to differences in individual learning ability and acceptance speed, some new mothers may need a longer time to master these skills. At the same time, faced with a large amount of nursing information from various channels, such as the internet and relatives, and friends, there may be contradictory content, making it difficult for new mothers to screen and master the correct self-care methods.

*Having mastered the key points of preventive care for mastitis, as well as the correct methods for identifying abnormal lochia and perineal care. Beyond that, I’m not too familiar with other details. (P1)*

*Not knowing how to regulate postpartum emotions. (P6)*

*The uterus is restored. I heard them (friends) say that they will come to the hospital after that, but I don’t know how long it will take to start, or what it is. (P9)*

*I don’t know a lot of the questions. (P10)*

*When I walk, I feel my abdomen shake, and I wonder why this happens. (P13)*

*I only know that the doctor said to change the wound dressing within 5 days. (P17)*

*Mastered a little bit of breastfeeding skills, but can’t massage the breast. (P18)*

#### Sub-Theme 3: Difficulties in Parenting

As primiparas, they lack practical parenting experience and are often difficult to predict and meet in the face of various needs of newborns (such as feeding, diaper changing, calming crying, etc.), and are easily overwhelmed and even frustrated.

*The skills for infant care are still rather lacking. (P6)*

*When he (the baby) cried, I felt a headache. (P7)*

*Breastfeeding is not yet skilled. (P9)*

*The ability to distinguish the baby’s crying sounds is insufficient, making it difficult to accurately determine their needs or the cause of their discomfort. (P12)*

*Do not know how often to feed. (P14)*

### Theme 4: Nursing Service Needs

#### Sub-Theme 1: Maternal and Infant Knowledge and Nursing Needs

From pregnancy to delivery, primiparas undergo significant changes in body systems. Postpartum women have an urgent need for knowledge and skills in self-rehabilitation and infant care, and they are very eager to get professional guidance from medical staff.

*It is hoped that the hospital can be equipped with professional lactation consultants. (P2)*

*I would like to know what uterine prolapse is and how to prevent it. (P4)*

*Want to learn the skills for identifying baby crying sounds in order to accurately determine the baby’s needs. (P12)*

*What does postpartum rehabilitation include? I’m confused about the online information regarding abdominal muscle separation and pelvic floor recovery. (P16)*

#### Sub-Theme 2: Continuing Nursing Knowledge Needs

Puerpera faces the triple pressure of physical rehabilitation, parenting, and role change after delivery. However, the limited learning during hospitalization, the confusion of online information, and the lack of health care services mean that there is an urgent need for medical staff to provide continuous postpartum guidance.

*Is there any online consultation or home follow-up service available after discharge? (P4)*

*I’m still a little worried about the post-homecare arrangements. In case of any emergencies, how can I contact professional guidance through which phone number? (P10)*

*Is there a rental service for home jaundice detection devices? (P13)*

### Theme 5: Interactive Participation

#### Sub-Theme 1: The Implementation of Interactive Assessments is Inadequate

The “indoctrination-based” health education and nursing model results in less interaction and feedback between nurses and patients, which is not conducive to the new mothers’ acquisition and understanding of relevant information about postpartum preparation. Moreover, there are differences in the understanding of health guidance content and its effects between the medical staff and the new mothers.

*The midwife only demonstrated the process of breast massage once, but it seems that I haven’t fully grasped it. (P2)*

*No one has assessed whether I’ve mastered these skills. (P7)*

*They only briefly asked if I needed help. (P14)*

*They checked some basics but said I need more practice. Honestly, I still have many unanswered questions. (P16)*

#### Sub-Theme 2: Insufficient Participation

Primiparas are weak and need to recuperate after childbirth, and most of them are only children with strong family support. Therefore, the care and health education of infants are often provided by family members so that infants can focus on their own recovery.

*It is the child’s father who should listen to the health education.I feel I am confused. Given a learning list, I can’t remember. (P2)*

*It is possible that they (family members) know the precautions, but I do not know. (P8)*

*I was given a discharge list. I’m not sure if there are any other precautions to follow. (P14)*

#### Sub-Theme 3: Expect to Participate in Decisionmaking

Some first-time mothers reported that discharge decisions were primarily determined by physicians without their full participation in related discussions. However, they expressed a desire to engage in shared decision-making regarding both the timing of discharge and post-discharge care instructions.

*I’d like to be involved in discharge planning discussions. I want to know in advance what documents or preparations are needed for the discharge procedures. (P3)*

*I want to participate—it helps me learn more about post-discharge care instructions and other important details. (P4)*

*I think healthcare providers should proactively ask for the patient’s input. Sometimes, our own experiences differ from your clinical assessments. (P7)*

## DISCUSSION

This study highlights dual psychological dynamics in primiparas’ discharge readiness: while eager to return home as a recovery milestone, they simultaneously experience role-transition anxiety and recovery uncertainty, aligning with Guo’s findings^(17)^. Similarly, a qualitative study on parents of children admitted to the intensive care unit found that although most parents subjectively thought that their children were ready for discharge when they were discharged from the hospital, their accompanying anxiety may reflect the gap between objective readiness and actual needs^(18)^. These findings underscore the necessity for clinicians to conduct comprehensive discharge readiness assessments and provide tailored guidance before discharge. Notably, during the interview process of this study, some participants reported that they were unaware of the specific criteria for discharge readiness and thus could not accurately assess whether they had met the necessary standards. Therefore, in the future, it is suggested to adopt specific standard tools that are suitable for assessing the readiness of pregnant and postpartum women for discharge. Comprehensive assessment should be conducted from multiple dimensions, such as physiology, psychology, and social support, to determine whether they are ready for discharge, in order to facilitate their smooth return to family and society. This aligns with the King’s TGA, which emphasizes nurse-patient communication, goal-setting, and collaborative care. By using the King’s TGA, healthcare providers can ensure mothers meet medical standards while actively setting personalized recovery goals.

This study reveals that when confronted with the dual challenges of postpartum recovery and infant care, some primiparas are able to actively mobilize various social resources to seek help. Comprehensive care support from partners, relatives, and others not only effectively alleviates the parenting pressure of primiparas but also creates a valuable window for their physical recovery. Social support is an important component for individuals to cope with stress and is closely related to the readiness for discharge^(19)^. Therefore, in addition to obtaining support from medical staff, strengthening the family-friend support system can enhance the positive coping ability of primiparas and improve their quality of life after discharge. The King’s framework emphasizes the role of social systems in goal attainment. Including family and community support in discharge planning reflects this, showing that maternal health goals depend on effective interaction between personal, interpersonal, and social systems.

This study identifies that primiparas, due to their lack of prior childbirth experience, commonly face insufficient health knowledge, inadequate care skills, and weak psychological adaptability, while still desiring ongoing professional support post-discharge. Relevant studies have pointed out that primiparas face many difficulties after discharge, including breastfeeding difficulties, parenting powerlessness, anxiety, body recovery distress, sleep deprivation, and lack of infant care knowledge^(20)^. Furthermore, fragmented postpartum care information and inconsistent perceptions between healthcare providers and patients, compounded by the passive, didactic traditional health education model, result in poor maternal comprehension of health instructions^(21)^. Research demonstrates that virtual reality (VR) technology, through immersive experiences, strong situational realism, and high interactivity, can help mothers intuitively grasp complex medical information while enabling real-time Q&A with providers, fostering collaborative engagement, and improving maternal-infant care knowledge acquisition^(22)^. Thus, future studies should harness AI, big data, and 5G technologies to revolutionize nursing interventions, delivering tailored health education that promotes bidirectional provider-patient engagement. Such innovations can empower primiparas’ self-management agency, augment their parental competence and recovery literacy, thereby optimizing discharge preparedness and adaptive capacity.

In China, postpartum home visitation systems are also limited by infrequent visits, loosely regulated staff qualifications, and inadequate attention to maternal mental health, failing to meet patients’ needs^(23)^. Studies confirm that “Internet+”-based home postpartum care models deliver sustained, high-quality support to effectively address postpartum health demands^(24)^. Therefore, in the future, healthcare professionals can provide continuous care support through “Internet +” nursing platforms and 24-hour midwife consultation hotlines, helping new mothers enhance their parenting skills and confidence in home rehabilitation.

Due to heavy clinical workloads, healthcare providers often lack sufficient time for nurse-patient communication or fail to adequately assess its impact, inadvertently limiting patient involvement in care decisions. However, some primiparas expressed willingness to participate in discharge planning discussions. Research shows that empowerment interventions for these women can enhance childbirth experiences, improve delivery outcomes, and increase maternal satisfaction^(25)^. Patient-centered nursing interactions promote self-management participation, allowing nurses to better evaluate disease knowledge and emergency preparedness. This facilitates tailored education that strengthens knowledge gaps, ultimately improving discharge guidance quality and readiness^(26)^. These findings collectively underscore the critical role of effective nurse-patient interactions in promoting maternal and infant health. Notably, studies suggest that patients’ limited medical knowledge hinders their ability to anticipate post-discharge needs^(27)^. Therefore, healthcare providers should adopt an active nurse-patient engagement model, guiding primiparas in discharge discussions and thoughtfully involving them in treatment decisions. This approach helps them better understand post-discharge challenges and care requirements. Such practices directly operationalize the King’s TGA by fostering mutual understanding, setting shared objectives, and evaluating progress toward agreed-upon health goals.

## LIMITATIONS AND FUTURE RESEARCH DIRECTIONS

This study offers insights into Chinese primiparas’ discharge readiness and postpartum care needs. However, as a qualitative inquiry, the findings are context-specific and primarily intended to enhance understanding within similar settings. Future research involving larger and more diverse samples could further explore the transferability of these insights, and the King’s TGA could guide the development of discharge readiness interventions.

## CONCLUSION

In current healthcare settings, the discharge readiness needs of primiparous women remain inadequately addressed. Healthcare providers should systematically evaluate multifactorial influences on postpartum discharge preparedness, address specific nursing requirements, enhance nurse-patient engagement, and promote maternal self-management competencies to facilitate post-discharge adaptation and achieve optimal maternal-infant health outcomes.

## DATA AVAILABILITY

The entire dataset supporting the results of this study was published in the article itself.
