# Supplementary material for: Perceptions of discharge readiness among primiparas based on the King’s theory of goal attainment: a qualitative study
Source: Rev Esc Enferm USP. 2025 Dec 15;59:e20250187. doi: 10.1590/1980-220X-REEUSP-2025-0187en (PMC12704598; doi:10.1590/1980-220X-REEUSP-2025-0187en)
Supplement: Translation

# Translation: Percepciones sobre la preparación para el alta hospitalaria entre las primíparas según la teoría del logro de metas de King: un estudio cualitativo

**DOI:** 10.1590/1980-220X-reeusp-2025-0187es

**Authors:** Hangcheng Liu, Hong Jiang, Miao Li, Xixi Li

**Affiliations:** 1 University of Electronic Science and Technology of China, School of Medicine, Mianyang Central Hospital, Department of Obstetrics and Gynecology, Mianyang, Sichuan Province, China.; 2 University of Electronic Science and Technology of China, School of Medicine, Mianyang Central Hospital, Department of Nursing, Mianyang, Sichuan Province, China.; 3 North Sichuan Medical College, College of Nursing, Nanchong, Sichuan Province, China.

**Keywords:** Paridad, Alta del Paciente, Relaciones Enfermero-Paciente

## Abstract

**Objetivo:**

Analizar las experiencias percibidas y las necesidades de enfermería no satisfechas de las primíparas con respecto a la preparación para el alta, utilizando la Teoría del Logro de Metas de King, para optimizar la gestión clínica del alta.

**Métodos:**

Utilizando el método fenomenológico en la investigación cualitativa, mediante un muestreo intencional, se realizaron entrevistas en profundidad semiestructuradas y cara a cara a 18 primíparas de un hospital de tercer nivel en la Provincia de Sichuan. Se utilizó el software NVivo 15.0 para gestionar los datos de las entrevistas y el método de análisis fenomenológico de 7 pasos de Colaizzi para analizar los datos.

**Resultados:**

Se identificaron un total de 5 temas principales y 12 subtemas, que incluyen: psicología compleja al alta (expectativas positivas, llenas de preocupaciones), factores promotores de la preparación para el alta (afrontamiento proactivo personal, alto grado de apoyo familiar), factores obstructivos de la preparación para el alta (conocimientos débiles sobre autocuidado, habilidades de autocuidado insuficientes, dificultades en la crianza), necesidades de servicio de enfermería (necesidades de conocimiento y cuidados materno-infantiles, necesidades de conocimiento sobre cuidados continuados) y participación interactiva (la implementación de evaluaciones interactivas es inadecuada, participación insuficiente, expectativa de participar en la toma de decisiones).

**Conclusión:**

Las necesidades de preparación para el alta de las primíparas siguen sin satisfacerse. El personal médico debe abordar los diversos factores que afectan la preparación para el alta, mejorar la interacción entre enfermeras y pacientes, y aumentar la iniciativa de las primíparas en el autocuidado, para ayudarlas a adaptarse a la vida después del alta y lograr los objetivos de salud materno-infantil.

## INTRODUCCIÓN

La Organización Mundial de la Salud (OMS) indica que en 2023 se produjeron aproximadamente 260 000 muertes maternas en todo el mundo durante el embarazo, el parto o el posparto, y que la mayoría de estas muertes eran evitables^(1)^, lo que pone de relieve la necesidad crucial de la seguridad materna. En 2021, la política china de optimización de la fertilidad para promover un desarrollo poblacional equilibrado a largo plazo hizo hincapié en la plena implementación del avance de los servicios integrados de atención posparto para las madres^(2)^. Al mismo tiempo, las reformas sanitarias de China y la adopción generalizada de protocolos de recuperación mejorados han transformado los modelos de atención perinatal^(3,4)^. Si bien benefician a las madres al permitirles una recuperación funcional más rápida, aliviar el dolor, acortar la estancia hospitalaria y reducir los costos, estos cambios conllevan el riesgo de un alta prematura antes de la recuperación fisiológica completa, lo que podría aumentar los retos de adaptación física y psicológica posparto^(5,6).^

La preparación para el alta hospitalaria (Readiness for Hospital Discharge - RHD), introducida por el académico británico Fenwick en 1979, implica una evaluación multidimensional (fisiológica, psicológica y funcional social) por parte de los proveedores de atención médica para determinar la capacidad de una paciente para pasar del hospital a su hogar^(7)^. Como elemento clave de la planificación del alta, la RHD se ha implementado ampliamente en las clínicas^(8)^. Las investigaciones confirman que una preparación óptima para el alta reduce los riesgos de reingreso, previene las complicaciones posteriores al alta y mejora la estabilidad fisiológica^(9)^. Una preparación insuficiente para el alta puede dar lugar a la falta de conocimientos y habilidades relevantes de las primíparas, a una falta temporal de capacidad para el autocuidado y el cuidado del bebé, por lo que es fácil que se produzcan ansiedad posparto, depresión y otros problemas psicológicos, lo que afectará a la rehabilitación materna e incluso afectará gravemente al crecimiento y desarrollo de los bebés^(6)^. Las primíparas son más vulnerables a las complicaciones del embarazo debido a que es su primera experiencia con la gestación y el parto. Factores como los conocimientos limitados sobre salud, la falta de habilidades de enfermería, la escasa adaptabilidad psicológica y las estancias hospitalarias más cortas contribuyen a que su riesgo sea mayor^(10)^.

La teoría del logro de metas de King (TLM) fue propuesta por King en 1981, que hace hincapié en la atención «centrada en el paciente» en el proceso de enfermería y anima a los pacientes a participar activamente en la atención sanitaria. A través del modelo cíclico de cuatro etapas de «interacción enfermera-paciente, sinergia de objetivos, medición y evaluación dinámica», se puede ayudar a los pacientes a alcanzar el mejor estado en un breve periodo de tiempo^(11)^. Este proceso demuestra las características sistemáticas y dinámicas de las actividades de enfermería, como se muestra en la Figura 1. Esta teoría se ha utilizado ampliamente en los campos de la gestión de enfermedades crónicas, la educación sanitaria, la enfermería psicológica y otros campos, y se ha demostrado que mejora los resultados de salud de los pacientes^(12)^.

**Figura 1:** Teoría del logro de metas de King.

Por lo tanto, basándose en la TLM de King y en el método de investigación fenomenológica en la investigación cualitativa, este estudio realizó entrevistas en profundidad a mujeres primíparas para aclarar su experiencia multidimensional percibida de la preparación para el alta, identificar sus necesidades de enfermería no satisfechas y proporcionar una base para que el personal médico y de enfermería clínico optimice la gestión de la preparación para el alta de las mujeres primíparas.

## MÉTODO

### Diseño y Entorno

Este estudio adoptó un diseño cualitativo. Se utilizó el método de investigación fenomenológica^(13)^ para analizar la percepción de las primíparas sobre la preparación para el alta y las necesidades de enfermería. Los resultados de este estudio se comunicaron utilizando las Normas Unificadas para la Presentación de Informes de Investigación Cualitativa (COREQ)^(14)^.

### Población, Criterios de Inclusión y Exclusión

Mediante un muestreo intencional, se seleccionó como participantes en el estudio a mujeres en posparto que acudieron a la sala de obstetricia de un hospital general terciario de grado A de la provincia de Sichuan entre abril y mayo de 2024. La estrategia de muestreo siguió el muestreo de máxima variación para garantizar la diversidad en múltiples dimensiones, incluyendo la edad materna, el nivel educativo, la ocupación y el lugar de residencia. Los criterios de inclusión de las madres fueron: 1) edad ≥ 20 años^(15)^; 2) primíparas; 3) estado posparto estable y alta sin complicaciones; 4) conciencia mental normal y buena capacidad de comunicación; 5) participación voluntaria y firma del consentimiento informado. Los criterios de exclusión fueron los siguientes: 1) comorbilidades/complicaciones graves antes o después del parto; 2) anomalías en los recién nacidos; 3) aquellas que se retiraron voluntariamente durante la entrevista.

### Entrevista y Procedimiento

Los investigadores revisaron la bibliografía relevante nacional e internacional, guiados por la TLM de King y combinando las sugerencias de expertos clínicos relevantes, el equipo de investigación (que incluía un supervisor de maestría, seis estudiantes de posgrado, un subdirector médico de obstetricia y una subdirectora de enfermería de obstetricia) elaboró inicialmente un esquema de entrevista. Se realizó una entrevista previa a tres primíparas que cumplían los criterios de inclusión y exclusión, y se revisó el esquema de la entrevista en función de los resultados de la misma. El esquema formal de la entrevista se muestra en la Cuadro 1.

**Cuadro 1:** Guía de entrevista – Mianyang, Sichuan, China, 2024.

Número	Preguntas
1	Mientras se prepara para salir del hospital, ¿puede describir cómo se siente en este momento? (incluido su estado físico y mental)
2	¿Qué ha aprendido sobre su propia rehabilitación y el cuidado del bebé para poder pasar con éxito del hospital a la atención domiciliaria?
3	¿Tuvo algún problema durante su estancia? En caso afirmativo, ¿cómo se resolvió?
4	¿Qué tipo de orientación le proporcionó la enfermera durante su hospitalización?
5	Durante los preparativos para el alta, ¿participó en el proceso de toma de decisiones? (por ejemplo, hora del alta, planificación de los cuidados, etc.) ¿Qué opina de este nivel de participación?
6	¿Tiene alguna sugerencia o comentario que le ayude a dar de alta del hospital a su casa?

Este estudio utilizó un enfoque fenomenológico para analizar las percepciones de las primíparas sobre su preparación para el alta y sus necesidades de atención. Antes de la recolección de datos, todos los investigadores recibieron formación sistemática en metodologías cualitativas. Con la presentación de obstetras o enfermeras encargadas, los investigadores establecieron una relación inicial con las posibles participantes, seguida de explicaciones detalladas sobre los objetivos del estudio, los procedimientos, las medidas de confidencialidad y los principios de participación voluntaria, que culminaron en el consentimiento informado por escrito. Las entrevistas se programaron dentro de las 24 horas previas al alta. Se seleccionó un espacio privado (habitación individual o unidad de cuidados centrada en la familia) de mutuo acuerdo para garantizar la confidencialidad. Las entrevistas semiestructuradas, con una duración media de 30 a 40 minutos, se grabaron en audio. Los investigadores se adhirieron a la guía de la entrevista, manteniendo al mismo tiempo la flexibilidad, practicando la escucha activa, documentando las señales no verbales (por ejemplo, expresiones faciales, cambios de tono) y evitando preguntas capciosas o interrupciones. Para la gestión de los datos, todos los materiales se anonimizaron (P1–P20) y el investigador principal los almacenó cifrados. La transcripción literal y el diario reflexivo se completaron en las 24 horas posteriores a la entrevista. La muestra final incluyó a 18 primíparas (reclutamiento inicial: 20; 2 se retiraron debido a las necesidades de cuidado de los bebés), y el muestreo continuó hasta la saturación de los datos.

### Alnállisis de Datos

En las 24 horas posteriores a la entrevista, el investigador transcribió rápidamente la grabación a datos de texto, complementó la información no verbal, como los movimientos y expresiones registrados durante la entrevista, y luego entregó los datos de la grabación y los datos de texto a otro miembro del equipo de investigación capacitado para su verificación, a fin de garantizar la integridad y exactitud de la transcripción. En este estudio, se utilizó el software NVivo 15.0 para gestionar los datos de texto y se utilizó el método de análisis fenomenológico de 7 pasos de Colaizzi^(16)^ para analizar los datos. Los pasos específicos son los siguientes: (1) Escuchar y leer repetidamente los materiales para recolectar, familiarizarse con todo el contenido y formarse una comprensión general; (2) Importar los materiales de texto de Word al software NVivo 15.0, leerlos palabra por palabra, identificar y extraer las declaraciones relevantes y significativas que están relacionadas con la pregunta de investigación; (3) Codificar las declaraciones repetitivas y significativas; (4) Resumir y categorizar los puntos de vista codificados y construir un marco temático; (5) Describir cada tema en detalle e insertar declaraciones originales típicas; (6) Identificar puntos de vista similares y determinar los temas; (7) Comunicar los resultados a los encuestados para verificar la autenticidad y exactitud del contenido.

### Aprobación Ética

El estudio se llevó a cabo en estricta conformidad con las directrices y normativas pertinentes y los principios de la Declaración de Helsinki. El estudio fue aprobado por el Comité de Ética Biomédica del Hospital Central de Mianyang, Universidad de Ciencia y Tecnología Electrónica de China, antes de su realización (ID: S20240310-02). Antes de la entrevista, el investigador informó detalladamente a las primíparas sobre el contenido del estudio y estas firmaron un consentimiento informado. Las transcripciones se anonimizaron sustituyendo los nombres por códigos (P1–P18). Las participantes podían retirarse del estudio en cualquier momento.

## RESULTADOS

### Características Demográficas de las participantes

De acuerdo con el principio de saturación de datos, se incluyó como entrevistadas a un total de 18 primíparas casadas que cumplían los criterios de inclusión y exclusión. De acuerdo con el principio de confidencialidad, el nombre de la entrevistada se sustituyó por un número, concretamente P1–P18. La información general de las entrevistadas se muestra en la Tabla 1.

**Tabla 1:** Información general sobre los entrevistados (n = 18) - Mianyang, Sichuan, China, 2024.

Número	Edad (años)	Grupos étnicos	Nivel educativo	Ocupaciones	Lugar de residencia	Ingresos per cápita (yuanes/mes)	Seguro médico	Comorbilidad	Modo de parto	Cuidador(es)
P1	24	Han	Escuela técnica superior	–	Población del condado	3000-5000	Nuevo sistema médico cooperativo rural	–	Parto natural	1
P2	28	Han	Licenciatura	Miembro del personal	Área urbana	>8000	Tratamiento médico para trabajadores	–	Cesárea	2
P3	28	Han	Escuela técnica superior	Miembro del personal	Área urbana	5000-8000	Tratamiento médico para trabajadores	–	Parto natural	1
P4	31	Han	Escuela secundaria técnica	–	Área urbana	5000-8000	Nuevo sistema médico cooperativo rural	DMG, CIE	Cesárea	2
P5	36	Qiang	Licenciatura	Funcionario público	Área urbana	8000	Tratamiento médico para trabajadores	Diabetes gestacional, hipertiroidismo	Cesárea	2
P6	28	Han	Escuela técnica superior	Profesor	Área urbana	3000-5000	Tratamiento médico para trabajadores	–	Cesárea	1
P7	27	Han	Escuela secundaria técnica	–	Área urbana	5000-8000	Nuevo sistema médico cooperativo rural	–	Cesárea	2
P8	34	Han	Licenciatura	Profesor	Área urbana	>8000	Tratamiento médico para trabajadores	–	Cesárea	2
P9	39	Han	Escuela técnica superior	Profesor	Área urbana	5000-8000	Tratamiento médico para trabajadores	–	Cesárea	1
P10	29	Han	Escuela técnica superior	Miembro del personal	Área urbana	5000-8000	Tratamiento médico para trabajadores	–	Cesárea	2
P11	33	Han	Máster	Enfermera	Área urbana	>8000	Tratamiento médico para trabajadores	Trombofilia	Cesárea	2
P12	27	Han	Licenciatura	Enfermera	Área urbana	>8000	Tratamiento médico para trabajadores	–	Parto natural	1
P13	36	Han	Secundaria	–	Municipio	3000-5000	Nuevo sistema médico cooperativo rural	–	Cesárea	1
P14	22	Han	Instituto	Miembro del personal	Municipio	3000-5000	Nuevo sistema médico cooperativo rural	–	Parto natural	1
P15	29	Han	Máster	Miembro del personal	Área urbana	>8000	Tratamiento médico para trabajadores	–	Cesárea	2
P16	27	Tujia	Escuela técnica superior	–	Población del condado	5000-8000	De su propio bolsillo	–	Parto natural	2
P17	29	Han	Licenciatura	Funcionario	Área urbana	5000-8000	Tratamiento médico para trabajadores	Hipotiroidismo	Cesárea	1
P18	26	Han	Instituto	–	Área urbana	5000-8000	Nuevo sistema médico cooperativo rural	DMG	Cesárea	2

Nota. DMC, diabetes mellitus gestacional; CIE, colestasis intrahepática del embarazo.

Se transcribieron literalmente dieciocho grabaciones y, finalmente, se formaron doce subtemas con conceptos similares mediante codificación manual, agrupación de temas y deliberación repetida. Se analizaron los doce subtemas y se resumieron en cinco temas: psicología compleja al alta, factores que favorecen la preparación para el alta, factores que dificultan la preparación para el alta, necesidades de servicios de enfermería y participación interactiva, como se muestra en la Cuadro 2.

**Cuadro 2:** Temas y subtemas clave – Mianyang, Sichuan, China, 2024.

Temas	Subtemas
Psicología compleja en el momento del alta	1. Expectativas positivas
	2. Lleno de preocupaciones
Factores que favorecen la preparación para el alta	1. Afrontamiento proactivo personal
	2. El grado de apoyo familiar era alto
Impedimentos para la preparación del alta	1. Conocimientos insuficientes sobre autocuidado
	2. Habilidades de autocuidado insuficientes
	3. Dificultades en la crianza de los hijos
Necesidades de servicios de enfermería	1. Conocimientos y necesidades de cuidados de la madre y el bebé
	2. Necesidades continuas de conocimientos de enfermería.
Participación interactiva	1. La implementación de evaluaciones interactivas es inadecuada
	2. Participación insuficiente
	3. Expectativa de participar en la toma de decisiones

### TEMA 1:Psicología Compleja al momento Del Alta

#### Subtema 1: Expectativas Positivas

Algunas primíparas creen que se han recuperado bien y que no tienen complicaciones posparto, y con el cuidado y el apoyo suficientes de sus familias, están ansiosas por salir del hospital. Otras, sin embargo, debido al entorno hospitalario relativamente desconocido y ruidoso, la falta de protección de la privacidady los horarios irregulares, están más ansiosas por regresar al entorno familiar y cómodo del hogar.

*Todo el proceso del parto fue bien (risas). Estoy feliz de irme a casa. (P1)*

*La sala era tan estrecha que era fácil que los bebés se afectaran entre sí, uno lloraba, el otro lloraba, y me preocupaba molestar a los demás. La casa es más espaciosa, por lo que las dos personas que acompañan la cama pueden turnarse para descansar, lo cual es más conveniente. (P5)*

*En el momento en que me dieron el alta del hospital, me sentí muy feliz, porque había estado despierta toda la noche y me sentía muy cansada. (P12)*

*El estado de ánimo es muy feliz, la cama de la sala es demasiado dura y el entorno es mejor después de volver a casa. (P17)*

#### Subtema 2: Lleno de Preocupaciones

Debido a la reducción de la estancia hospitalaria y a ser madres primerizas, algunas primíparas se preocupan principalmente por la mala recuperación tras el alta, la insuficiente capacidad para cuidar al recién nacido, la falta de apoyo médico profesional y las preocupaciones sobre los posibles riesgos.

*Preocupada por cómo llegar a casa, con miedo a caminar porque la herida todavía le duele mucho (frunce el ceño). El bebé estaba en la unidad de cuidados y me preocupaba la lactancia materna porque mis pechos se hinchaban fácilmente y no sabía qué hacer. (P2)*

*Me preocupa no poder cuidar bien al bebé cuando llegue a casa. (P6)*

*Me preocupa si tendré problemas para defecar cuando llegue a casa. También me preocupa que la ictericia del bebé no sea normal y que la alimentación sea difícil. (P12)*

*Siento que la herida todavía me duele bastante y me da miedo que no se recupere bien. (P18)*

### Tema 2: Factores Que Favorecen La Preparación Para El Alta

#### Subtema 1: Afrontamiento Proactivo Personal

En la era de la información, con la ayuda de la inteligencia en red, las puérperas toman la iniciativa de obtener apoyo para el cuidado materno e infantil consultando al personal médico, uniéndose a grupos de madres, buscando conocimientos sobre la crianza de los hijos y leyendo libros especializados.

*Con la orientación de la partera, aprendí a amamantar. (P3)*

*Aprendí vídeos de rehabilitación, como los de los músculos del suelo pélvico y los músculos rectos abdominales, en TikTok. También asistí a un grupo de comunicación sobre maternidad en el hospital para aprender sobre la elección de pañales y cómo manejar el eccema y los vómitos de mi bebé. (P4)*

*Compré un libro sobre la dieta posparto para estudiarlo. (P10)*

*Si tengo algún problema, puedo preguntar a mis amigas que han pasado por situaciones similares y aprender de sus experiencias. (P15)*

#### Subtema 2: El Grado de Apoyo Familiar Fue Alto

En la etapa especial de la nueva maternidad, las primíparas recibieron un apoyo y cuidados integrales por parte de sus parejas, amigos y familiares, lo que no solo redujo su carga parental, sino que también les permitió disponer de más tiempo para descansar y recuperarse, mejorando así su sensación de competencia parental y preparándolas para los diversos retos que les esperaban tras el alta.

*Estoy de buen humor, ellos (mi esposo y mi madre) lo cuidan muy bien (al bebé), han estado estudiando estos días, creo que lo harán muy bien. (P1)*

*En cuanto a los bebés, mi hermana tiene dos hijos. Tanto mi hermana como mi madre tienen suficiente experiencia para saber cómo cuidar y alimentar a los bebés, y cómo recuperarme y qué precauciones tomar. (P2)*

*El papá está aprendiendo a observar, bañar y tocar al bebé para detectar la ictericia. (P6)*

### Tema 3: Obstáculos Para La Preparación Del Alta

#### Subtema 1: Los Conocimientos Sobre Autocuidado Eran Escasos

Debido a la influencia de los conceptos modernos de crianza, los servicios convenientes y el apoyo familiar, algunas primíparas tienen un conocimiento insuficiente de la complejidad de la recuperación posparto y el cuidado infantil, lo que puede generar fácilmente dependencia, poca disposición al autoaprendizaje y, en general, falta de conocimientos sobre la rehabilitación posparto y el cuidado infantil.

*Una vez que regreso a casa, mi familia se encarga de todo, así que yo solo tengo que concentrarme en descansar. (P1)*

*No tengo ninguna experiencia. Voy al centro de atención posparto para aprender. (P9)*

*Gracias a que contraté a mi doula posparto, ahora tengo mucha fuerza de voluntad, después de ir a casa a estudiar poco a poco. (P12)*

#### Subtema 2: Habilidades de Autocuidado Insuficientes

Las mujeres posparto se encuentran en un periodo de recuperación, y el cansancio y las molestias limitan su tiempo y energía para aprender habilidades de autocuidado. Además, debido a las diferencias en la capacidad de aprendizaje y la velocidad de aceptación de cada persona, algunas madres primerizas pueden necesitar más tiempo para dominar estas habilidades. Al mismo tiempo, ante la gran cantidad de información sobre lactancia que reciben a través de diversos canales, como Internet y sus familiares y amigos, puede haber contenidos contradictorios, lo que dificulta a las madres primerizas seleccionar y dominar los métodos correctos de autocuidado.

*He dominado los puntos clave del cuidado preventivo de la mastitis, así como los métodos correctos para identificar loquios anormales y el cuidado perineal. Más allá de eso, no estoy muy familiarizada con otros detalles. (P1)*

*No saber cómo regular las emociones posparto. (P6)*

*El útero se recupera. Les oí (a mis amigas) decir que vendrán al hospital después de eso, pero no sé cuánto tiempo tardará en empezar ni en qué consiste. (P9)*

*No sé muchas cosas. (P10)*

*Cuando camino, siento que me tiembla el abdomen y me pregunto por qué ocurre esto. (P13)*

*Solo sé que el médico dijo que había que cambiar el vendaje de la herida en un plazo de 5 días. (P17)*

*He aprendido un poco sobre la lactancia materna, pero no sé cómo dar masajes en los senos. (P18)*

#### Subtema 3: Dificultades en La Crianza de Los Hijos

Como madres primerizas, carecen de experiencia práctica en la crianza de los hijos y, a menudo, les resulta difícil predecir y satisfacer las diversas necesidades de los recién nacidos (como la alimentación, el cambio de pañales, calmar el llanto, etc.), y se sienten fácilmente abrumadas e incluso frustradas.

*Las habilidades para el cuidado del bebé son aún bastante deficientes. (P6)*

*Cuando él (el bebé) lloraba, me dolía la cabeza. (P7)*

*Aún no domino la lactancia materna. (P9)*

*La capacidad para distinguir los sonidos del llanto del bebé es insuficiente, lo que dificulta determinar con precisión sus necesidades o la causa de su malestar. (P12)*

*No sé con qué frecuencia alimentarlo. (P14)*

### Tema 4: Necesidades De Servicios De EnfermeríA

#### Subtema 1: Conocimientos Y Necesidades de EnfermeríA de La Madre y El Bebé

Desde el embarazo hasta el parto, las primíparas experimentan cambios significativos en sus sistemas corporales. Las mujeres en el posparto tienen una necesidad urgente de conocimientos y habilidades para la autorrehabilitación y el cuidado del bebé, y están muy interesadas en recibir orientación profesional del personal médico.

*Se espera que el hospital cuente con asesores profesionales en lactancia. (P2)*

*Me gustaría saber qué es el prolapso uterino y cómo prevenirlo.(P4)*

*Quiero aprender a identificar los sonidos del llanto del bebé para determinar con precisión sus necesidades. (P12)*

¿Qué incluye la rehabilitación posparto? Estoy confundida con la información que encuentro en Internet sobre la separación de los músculos abdominales y la recuperación del suelo pélvico. (P16)

#### Subtema 2: Necesidades de Conocimientos de Enfermería Continuos

Las puérperas se enfrentan a la triple presión de la rehabilitación física, la crianza de los hijos y el cambio de roles después del parto. Sin embargo, el aprendizaje limitado durante la hospitalización, la confusión de la información en línea y la falta de servicios de salud hacen que sea urgente que el personal médico proporcione orientación posparto continua.

¿Existe algún servicio de consulta en línea o de seguimiento a domicilio después del alta? (P4)

*Todavía me preocupa un poco la organización de los cuidados posteriores al alta. En caso de emergencia, ¿cómo puedo contactar con un profesional para que me oriente? ¿A qué número de teléfono debo llamar? (P10)*

¿Existe algún servicio de alquiler de dispositivos para la detección de ictericia en el hogar? (P13)

### Tema 5: Participación Interactiva

#### Subtema 1: La Implementación De Evaluaciones Interactivas Es Inadecuada

El modelo de educación sanitaria y enfermería «basado en el adoctrinamiento» da lugar a una menor interacción y retroalimentación entre las enfermeras y las pacientes, lo que no favorece la adquisición y comprensión por parte de las madres primerizas de la información relevante sobre la preparación posparto. Además, existen diferencias en la comprensión del contenido de la orientación sanitaria y sus efectos entre el personal médico y las madres primerizas.

*La partera solo me enseñó una vez el proceso del masaje mamario, pero parece que no lo he comprendido del todo. (P2)*

*Nadie ha evaluado si he dominado estas habilidades. (P7)*

*Solo me preguntaron brevemente si necesitaba ayuda. (P14)*

*Comprobaron algunos aspectos básicos, pero dijeron que necesitaba más práctica. Sinceramente, todavía tengo muchas preguntas sin respuesta. (P16)*

#### Subtema 2: Participación Insuficiente

Las primíparas están débiles y necesitan recuperarse después del parto, y la mayoría de ellas son hijas únicas con un fuerte apoyo familiar. Por lo tanto, el cuidado y la educación sanitaria de los bebés suelen correr a cargo de los familiares, para que las madres puedan centrarse en su propia recuperación.

*Es el padre del niño quien debe escuchar la educación sanitaria, me siento confundida. Me dan una lista de cosas que aprender y no soy capaz de recordarlas. (P2)*

*Es posible que ellos (los familiares) conozcan las precauciones, pero yo no las conozco. (P8)*

*Me dieron una lista de alta. No estoy segura de si hay otras precauciones que deba seguir. (P14)*

#### Subtema 3: Esperanza De Participar En La Toma De Decisiones

Algunas madres primerizas informaron de que las decisiones sobre el alta las tomaban principalmente los médicos sin su plena participación en las discusiones relacionadas. Sin embargo, expresaron su deseo de participar en la toma de decisiones compartida tanto sobre el momento del alta como sobre las instrucciones de cuidados posteriores al alta.

*Me gustaría participar en las discusiones sobre la planificación del alta. Quiero saber con antelación qué documentos o preparativos se necesitan para los trámites del alta. (P3)*

*Quiero participar, ya que me ayuda a aprender más sobre las instrucciones de cuidados posteriores al alta y otros detalles importantes. (P4)*

*Creo que los profesionales sanitarios deberían pedir de forma proactiva la opinión del paciente. A veces, nuestras propias experiencias difieren de sus evaluaciones clínicas. (P7)*

## DISCUSIÓN

Este estudio destaca la doble dinámica psicológica en la preparación para el alta de las primíparas: aunque están ansiosas por volver a casa como hito de su recuperación, al mismo tiempo experimentan ansiedad por la transición de roles e incertidumbre sobre la recuperación, lo que coincide con las conclusiones de Guo^(17)^. De manera similar, un estudio cualitativo sobre padres de niños ingresados en la unidad de cuidados intensivos reveló que, aunque la mayoría de los padres pensaban subjetivamente que sus hijos estaban listos para el alta cuando salían del hospital, su ansiedad acompañante puede reflejar la brecha entre la preparación objetiva y las necesidades reales^(18)^. Estos hallazgos subrayan la necesidad de que los médicos realicen evaluaciones exhaustivas de la preparación para el alta y proporcionen orientación personalizada antes del alta. Cabe destacar que, durante el proceso de entrevistas de este estudio, algunos participantes informaron de que desconocían los criterios específicos para la preparación para el alta y, por lo tanto, no podían evaluar con precisión si cumplían los estándares necesarios. Por lo tanto, se sugiere adoptar en el futuro herramientas estándar específicas que sean adecuadas para evaluar la preparación de las mujeres embarazadas y puérperas para el alta. Se debe realizar una evaluación exhaustiva desde múltiples dimensiones, como la fisiología, la psicología y el apoyo social, para determinar si están listas para el alta, con el fin de facilitar su regreso sin contratiempos a la familia y la sociedad. Esto se ajusta a la TLM de King, que hace hincapié en la comunicación entre el personal de enfermería y los pacientes, el establecimiento de objetivos y la atención colaborativa. Mediante el uso de la TLM de King, los proveedores de atención médica pueden garantizar que las madres cumplan con los estándares médicos, al tiempo que establecen activamente objetivos de recuperación personalizados.

Este estudio revela que, cuando se enfrentan al doble reto de la recuperación posparto y el cuidado del bebé, algunas primíparas son capaces de movilizar activamente diversos recursos sociales para buscar ayuda. El apoyo integral de la pareja, los familiares y otras personas no solo alivia eficazmente la presión de la crianza de los hijos de las primíparas, sino que también crea una valiosa oportunidad para su recuperación física. El apoyo social es un componente importante para que las personas hagan frente al estrés y está estrechamente relacionado con la preparación para el alta^(19)^. Por lo tanto, además de obtener el apoyo del personal médico, el fortalecimiento del sistema de apoyo familiar y de amigos puede mejorar la capacidad de afrontamiento positivo de las primíparas y mejorar su calidad de vida después del alta. La teoría de King enfatiza el papel de los sistemas sociales en la consecución de objetivos. La inclusión del apoyo familiar y comunitario en la planificación del alta refleja esto, lo que demuestra que los objetivos de salud materna dependen de la interacción efectiva entre los sistemas personales, interpersonales y sociales.

Este estudio identifica que las primíparas, debido a su falta de experiencia previa en el parto, suelen enfrentarse a un conocimiento insuficiente sobre salud, habilidades de cuidado inadecuadas y una débil adaptabilidad psicológica, al tiempo que siguen deseando recibir apoyo profesional continuo después del alta. Estudios relevantes han señalado que las primíparas se enfrentan a muchas dificultades después del alta, entre ellas dificultades para amamantar, impotencia parental, ansiedad, angustia por la recuperación corporal, privación del sueño y falta de conocimientos sobre el cuidado del bebé^(20)^. Además, la información fragmentada sobre la atención posparto y las percepciones inconsistentes entre los proveedores de atención de salud y las pacientes, agravadas por el modelo tradicional de educación sanitaria pasivo y didáctico, dan lugar a una mala comprensión de las instrucciones sanitarias por parte de las madres^(21)^. Las investigaciones demuestran que la tecnología de realidad virtual (RV), a través de experiencias inmersivas, un fuerte realismo situacional y una alta interactividad, puede ayudar a las madres a comprender de forma intuitiva información médica compleja, al tiempo que permite realizar preguntas y respuestas en tiempo real con los proveedores, fomentando la colaboración y mejorando la adquisición de conocimientos sobre el cuidado materno-infantil^(22)^. Por lo tanto, los estudios futuros deberían aprovechar la inteligencia artificial, los macrodatos y las tecnologías 5G para revolucionar las intervenciones de enfermería, ofreciendo una educación sanitaria personalizada que promueva la participación bidireccional entre el proveedor y el paciente. Estas innovaciones pueden potenciar la capacidad de autogestión de las primíparas, aumentar su competencia parental y sus conocimientos sobre la recuperación, optimizando así la preparación para el alta y la capacidad de adaptación.

En China, los sistemas de visitas domiciliarias posparto también se ven limitados por la escasa frecuencia de las visitas, la escasa regulación de las cualificaciones del personal y la atención inadecuada a la salud mental materna, lo que no satisface las necesidades de las pacientes^(23)^. Los estudios confirman que los modelos de atención posparto a domicilio basados en «Internet+» ofrecen un apoyo sostenido y de alta calidad para abordar eficazmente las necesidades de salud posparto^(24)^. Por lo tanto, en el futuro, los profesionales de la salud podrán proporcionar apoyo continuo a través de plataformas de enfermería «Internet +» y líneas telefónicas de consulta con parteras las 24 horas del día, ayudando a las nuevas madres a mejorar sus habilidades parentales y su confianza en la rehabilitación en el hogar.

Debido a la elevada carga de trabajo clínico, los proveedores de atención médica a menudo carecen de tiempo suficiente para la comunicación entre enfermeras y pacientes o no evalúan adecuadamente su impacto, lo que limita inadvertidamente la participación de las pacientes en las decisiones sobre la atención. Sin embargo, algunas primíparas expresaron su disposición a participar en las discusiones sobre la planificación del alta. Las investigaciones muestran que las intervenciones de empoderamiento para estas mujeres pueden mejorar las experiencias del parto, mejorar los resultados del parto y aumentar la satisfacción materna^(25)^. Las interacciones de enfermería centradas en el paciente promueven la participación en el autocuidado, lo que permite a las enfermeras evaluar mejor los conocimientos sobre la enfermedad y la preparación para emergencias. Esto facilita una educación personalizada que refuerza las lagunas de conocimiento y, en última instancia, mejora la calidad y la preparación de la orientación para el alta^(26)^. Estos hallazgos subrayan colectivamente el papel fundamental de las interacciones eficaces entre enfermeras y pacientes en la promoción de la salud materna e infantil. En particular, los estudios sugieren que los conocimientos médicos limitados de los pacientes dificultan su capacidad para anticipar las necesidades tras el alta^(27)^. Por lo tanto, los proveedores de atención de salud deben adoptar un modelo de participación activa entre enfermeras y pacientes, guiando a las primíparas en las conversaciones sobre el alta e involucrándolas cuidadosamente en las decisiones sobre el tratamiento. Este enfoque les ayuda a comprender mejor los retos y las necesidades de atención después del alta. Estas prácticas ponen en práctica directamente la TLM de King, ya que fomentan el entendimiento mutuo, establecen objetivos comunes y evalúan el progreso hacia las metas de salud acordadas.

## LIMITACIONES Y DIRECCIONES DE INVESTIGACIÓN FUTURAS

Este estudio ofrece información sobre la preparación para el alta y las necesidades de cuidados posparto de las primíparas chinas. Sin embargo, al tratarse de una investigación cualitativa, los resultados son específicos del contexto y su objetivo principal es mejorar la comprensión en entornos similares. Las investigaciones futuras, con muestras más amplias y diversas, podrían explorar más a fondo la transferibilidad de estos conocimientos, y la TLM de King podría orientar el desarrollo de intervenciones para la preparación para el alta.

## CONCLUSIÓN

En los entornos sanitarios actuales, las necesidades de preparación para el alta de las primíparas siguen sin abordarse de manera adecuada. Los proveedores de atención médica deben evaluar sistemáticamente las influencias multifactoriales en la preparación para el alta posparto, abordar los requisitos específicos de enfermería, mejorar la participación de las enfermeras y las pacientes, y promover las competencias de autogestión materna para facilitar la adaptación después del alta y lograr resultados óptimos de salud materno-infantil.

## DISPONIBILIDAD DE DATOS

Todo el conjunto de datos que apoya los resultados de este estudio fue publicado en el propio artículo.
